# 

**Published:** 1903-06

**Authors:** 


*THE BRISTOL MEDICO-CHIRURGICAL SOCIETY.*

### CONTENTS.

| | PAGE |
|---|---|
| **Mobility.** By JOHN CHIENE, C.B., LL.D., F.R.S.E., M.D. | 97 |
| **The Influence of the Auricles on the Percussion of the Heart.** By D. R. PATERSON, M.D., M.R.C.P. | 110 |
| **Some Remarks upon the Finsen Light Treatment of Lupus.** By W. KENNETH WILLS, M.A., M.B., B.C. Cantab., M.R.C.S. | 119 |
| **On the Diagnosis of Malignant Disease of the Body of the Uterus, with Notes of a Case treated twice by Curettage, and finally by Hysterectomy.** By ERNEST W. HEY GROVES, M.D., B.Sc. Lond. | 128 |
| **Missing Links in Joint Disease.** By PRESTON KING, B.A., M.D., B.C. Cantab. | 137 |
| **Progress of the Medical Sciences:** | |
| **Medicine.** G. PARKER, M.D., M.A. Cantab. | 143 |
| **Surgery.** C. A. MORTON | 148 |
| **Obstetrics and Gynæcology.** WALTER C. SWAYNE, M.D. Lond. | 153 |
| **Reviews of Books** | 158 |
| **Editorial Notes** | 165 |
| **Notes on Preparations for the Sick** | 173 |
| **The Library of the Bristol Medico-Chirurgical Society** | 176 |
| **Meetings of Societies** | 179 |
| **Obituary Notices** | 187 |
| **Local Medical Notes** | 192 |

